# Polymorphisms of the *HRG*, *FETUB*, and *GUCY1A1* genes and their association with litter size in sheep

**DOI:** 10.5194/aab-67-153-2024

**Published:** 2024-04-11

**Authors:** Zizhen Ren, Xiaoyun He, Xiangyu Wang, Mingxing Chu

**Affiliations:** State Key Laboratory of Animal Biotech Breeding, Institute of Animal Science, Chinese Academy of Agricultural Sciences (CAAS), Beijing 100193, China

## Abstract

Litter size is one of the key factors affecting the efficiency of sheep breeding, and previous studies found that the *HRG*, *FETUB*, and *GUCY1A1* genes were closely related to litter size in sheep. This experiment aims to explore the polymorphisms of the g.405442728A > G locus of the *HRG* gene, the g.421655951C > T locus of the *FETUB* gene, and the g.414050897G > C locus of the *GUCY1A1* gene and their association with sheep litter size. The MassARRAY^®^ single-nucleotide polymorphism (SNP) genotyping technique was used to detect the polymorphisms of these loci in five sheep breeds, i.e., Small-tailed Han sheep, Hu sheep, Cele black sheep, Sunite sheep, and Bamei mutton sheep. In addition, the association between the polymorphisms of these genes and the litter size of Small-tailed Han sheep was also analyzed. The results showed that the g.405442728A > G locus of the *HRG* gene was moderately polymorphic (0.25 < PIC <0.5 ) in both monotocous and polytocous sheep breeds; the g.421655951C > T locus of the *FETUB* gene was lowly polymorphic (PIC <0.25 ) in five sheep breeds; the g.414050897G > C locus of *GUCY1A1* showed moderately polymorphism in Small-tailed Han sheep (0.25 ≤ PIC <0.5 ) and low polymorphism in four other sheep breeds (PIC <0.25 ). The chi-squared test results showed that the g.405442728A > G locus of the *HRG* gene was in the Hardy–Weinberg equilibrium state in five sheep breeds ( P>0.05 ). The g.421655951 C > T locus of the *FETUB* gene and the g.414050897G > C locus of the *GUCY1A1* gene were in the Hardy–Weinberg equilibrium state in Small-tailed Han sheep ( P>0.05 ) and in the Hardy–Weinberg disequilibrium state in other sheep breeds ( P<0.05 ). The association analysis showed that the g.405442728A > G locus of the *HRG* gene and the g.421655951C > T locus of the *FETUB* gene had a significant impact on the litter size of sheep ( P<0.05 ), while the g.414050897G > C locus of the *GUCY1A1* gene had no significant impact on the litter size ( P>0.05 ). In summary, the *HRG* gene and the *FETUB *gene can be used as potential molecular markers for the selection of the litter size in sheep.

## 1 Introduction

Fecundity is one of the most important economic traits of sheep. Sheep with high fecundity showed 2 to 3 times the lamb production efficiency and economic benefits compared with sheep with low fecundity. The premise of improving fertility is successful fertilization, but many factors can lead to fertilization failure. For example, zona pellucida sclerosis occurs during the spontaneous maturation of mouse oocytes (Felici et al., 1985), and sperm penetration can be affected by its sclerosis, which will prevent fertilization (Schroeder et al., 1990). A complete ovary is also a prerequisite for improving fertility, and blood vessels are closely related to the integrity of the ovary (McFee and Cupp, 2013). Few sheep show the characteristics of high fecundity and perennial estrus around the world, and the researchers found the main genes affecting fecundity in a small number of high-fecundity sheep breeds. It has been proven that the formation and changes in endometrial blood vessels may lead to early-pregnancy abortion (Lash et al., 2012; Banerjee et al., 2013). However, there are far more than a few genes that affect fecundity, and new genes need to be discovered constantly.

Histidine-rich glycoprotein (*HRG*) is composed of 75 kDa glycoprotein, which is synthesized in the liver and can be circulated in the plasma. It was firstly isolated from human serum in 1972 (Haupt and Heimburger, 1972) and then found in the plasma of rats, rabbits, chicken, cattle, and other vertebrates. *HRG* is usually classified as a member of the cysteine protease inhibitor (CPI), although some researchers suggested that *HRG* should be classified as a new family in the cystatin superfamily (Koide and Odani, 1987). The exact biological molecular function of *HRG* is still unclear, but *HRG* has a prominent histidine-rich domain, which may be the basis of the interaction between *HRG* and many molecules, affecting neutrophils, red blood cells, and vascular endothelial cells (Nishibori, 2022). Some research results show that the C633T polymorphism of the *HRG* gene may have an impact on ovarian reactivity, oocyte quality, and the development of fertilized eggs in Chinese women (Jin et al., 2015) and play an important role in recurrent abortion (Lindgren et al., 2013). A single-nucleotide polymorphism (SNP) (A1042G) of the *HRG* gene is also related to recurrent abortion (Elenis et al., 2014). *HRG* plays an important role in angiogenesis, immune function, and coagulation processes, which are inextricably linked to pregnancy (Elenis et al., 2014). In addition, the *HRG* gene has at least 10 SNPs (UniProt Consortium, 2014), among which C633T may have an impact on the fecundity of sheep.

*FETUB* is a novel liver cytokine that belongs to the cystatin superfamily, which, like the *HRG* gene, belongs to the cystatin cysteine protease inhibitor superfamily (Jung et al., 2015; Meex et al., 2015). The potential functions of *FETUB* have not been clearly explained, but it has some similarities to fetuin. *FETUB* can play a role before fertilization, making the zona pellucida harden only early (Dietzel et al., 2013; Stoecker et al., 2014), maintaining the permeability of the zona pellucida, and playing an important role in the fertilization process. After fertilization, a series of changes will cause the zona pellucida to harden (Burkart et al., 2012). Reversible infertility of female mice can be achieved by regulating *FETUB* (Floehr et al., 2017). Some studies have shown that *FETUB* has a certain expression in rodents and primates during the second trimester of pregnancy (Olivier et al., 2000). Sperm proteins are very important in oocyte maturation, fertilization, and early embryonic development (Binsila et al., 2021). *GUCY1A1* is a gene encoding soluble guanylate cyclase (sGC). In goats, sheep (Zhu et al., 2020), pigs (Roca et al., 2020), and other animals, *GUCY1A1* affects coronary artery disease (Kessler et al., 2017) and hypertension (Curtis, 2021), but these influences may change with other factors such as people's age (Malinowski et al., 2022a). *GUCY1A1* also affects platelet adhesion and thrombosis (Malinowski et al., 2022a).

To further understand the effects of the above three genes on sheep fecundity, Small-tailed Han sheep, Hu sheep, Cele black sheep, Sunite sheep, and Bamei mutton sheep were selected for this study. We screened SNPs in the above five sheep breeds and genotyped them using the methylation mass spectrometry^®^ platform. The distribution of each genotype in each sheep population was obtained, and the SNP loci related to reproductive traits were expected to provide valuable genetic markers for sheep genetic breeding.

## 2 Material and methods

### 2.1 Animals

Five sheep breeds were used in this experiment, i.e., 384 Small-tailed Han sheep, 96 Hu sheep, 96 Cele black sheep, 96 Sunite sheep, and 96 Bamei mutton sheep (Table 1). Blood was collected from the jugular vein and anticoagulated with glucose citrate at -20 °. At the same time, the litter size of Small-tailed Han sheep was recorded.

**Table 1:** Basic information of the sample.

Breed	Number	Type	Region
Small-tailed Han sheep	384	Polytocous	Yuncheng County, Shandong Province, China
Hu sheep	96	Polytocous	Xuzhou, Jiangsu Province, China
Cele black sheep	96	Polytocous	Cele County, Xinjiang Uygur Autonomous Region, China
Sunite sheep	96	Monotocous	Urad Front Banner, Bayannur, China
Bamei mutton sheep	96	Monotocous	Linhe District, Bayannur, China

### 2.2 Genotyping

Genotyping of the g.405442728 A > G locus of *HRG*, the g.421655951 C > T locus of *FETUB*, and the g.414050897 G > C locus of *GUCY1A1* in polytocous and monotocous sheep breeds was identified by the MassARRAY^®^ SNP genotyping technique.

### 2.3 Data analysis

Microsoft Excel 2021 software was used to count the genotype frequency, gene frequency, polymorphism information content (PIC), heterozygosity (He), and effective allele number (Ne). The Hardy–Weinberg equilibrium test was also conducted. A linear model, $y_{ijn}=\mu +P_{i}+G_{j}+I_{PG}+e_{ijn}$ , was applied to analyze the association of genotypes with litter size as in our previous study (He et al., 2019). Prediction of protein secondary structure was conducted by online tools: http://rna.tbi.univie.ac.at//cgi-bin/RNAWebSuite/RNAfold.cgi (last access: 18 September 2023​​​​​​​).

## 3 Results

### 3.1 Analysis of *HRG*, *FETUB*, and *GUCY1A1* polymorphism

**Figure 1:**
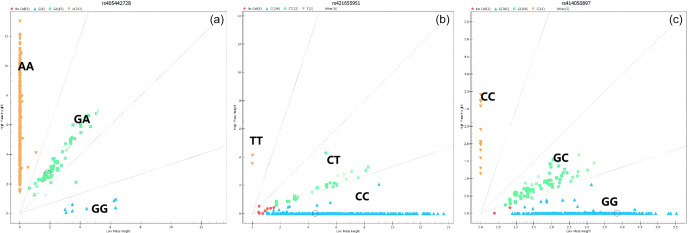
The genotyping results of the *HRG*, *FETUB*, and *GUCY1A1* genes. (Note: a – the g.405442728A > G locus representing the *HRG* gene; b – the g.421655951C > T locus representing the *FETUB* gene; c – the g.414050897G > A locus representing the *GUCY1A1* gene).

The results showed that the *HRG*, *FETUB*, and *GUCY1A1* genes all have three genotypes in Small-tailed Han sheep (Fig. 1).

**Figure 2:**
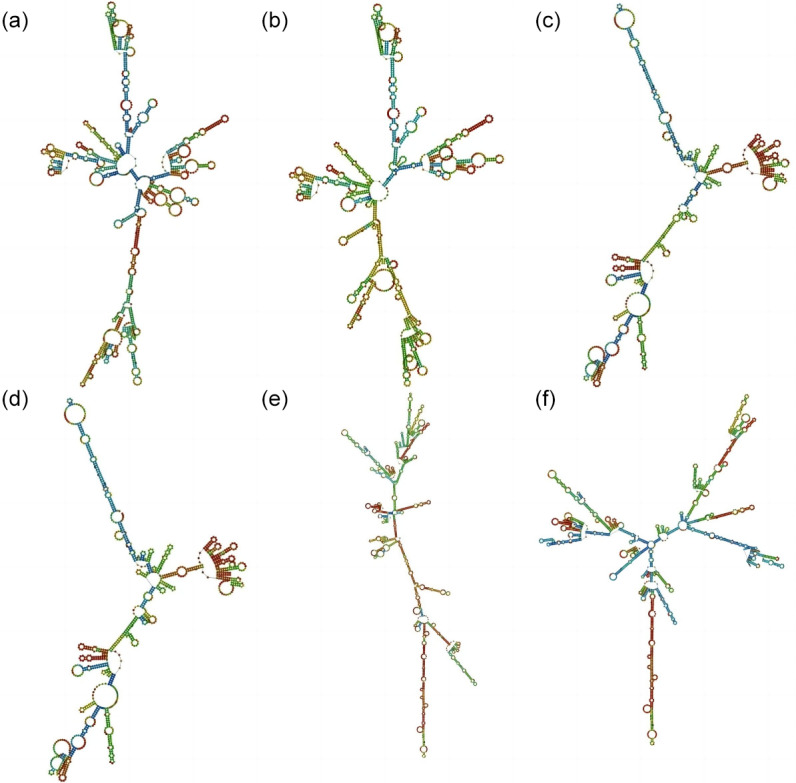
Secondary structures before and after gene mutation. **(a)**–**(b)** The secondary structure of *HRG* before and after mutation. **(c)**–**(d)** The secondary structure of *FETUB* before and after mutation. **(e)**–**(f)** The secondary structure of *GUCY1A1* before and after mutation. The different colors represent different base-pairing probabilities, where red > green > blue.

The genotypic frequencies of the g.405442728 A > G locus of the *HRG* gene and the g.421655951 C > T locus of the *FETUB* gene were significantly different between polytocous and monotocous sheep breeds ( P<0.01 ), and there were no significant differences in allele frequencies ( P>0.05 ). The dominant alleles in sheep breeds were A and C, respectively. The genotype frequency and allele frequency of the g.414050897 G > C locus of the *GUCY1A1* gene were significantly different between polytocous and monotocous sheep breeds ( P<0.01 ), and the dominant allele in polytocous and monotocous sheep breeds was G (Fig. 2).

**Table 2:** Genotypic and allelic frequencies of *HRG*, *FETUB*, and *GUCY1A1* polymorphism in polytocous and monotocous sheep breeds.

Locus	Genotype	Polytocous genotype	Monotocous genotype	χ2	Allele	Polytocous allele	Monotocous allele	χ2
		frequency	frequency	( p value)		frequency	frequency	( p value)
*HRG*	GG	0.08	0.06	0.00	G	0.18	0.21	0.24
g.405442728A > G	GA	0.22	0.30		A	0.82	0.79	
	AA	0.70	0.64					
*FETUB*	CC	0.90	0.90	0.00	C	0.95	0.95	0.92
g.421655951C > T	CT	0.09	0.10		T	0.05	0.05	
	TT	0.01	0.00					
*GUCY1A1*	GG	0.42	0.97	0.00	G	0.70	0.98	0.00
g.414050897G > C	GC	0.56	0.03		C	0.30	0.02	
	CC	0.02	0.00					

It can be seen from Table 3 that the g.405442728A > G locus of the *HRG* gene showed moderate polymorphism in Small-tailed Han sheep, Hu sheep, Cele black sheep, Sunite sheep, and Bamei mutton sheep ( 0.25< PIC <0.5 ). The g.421655951C > T locus of the *FETUB* gene showed low polymorphism in five sheep breeds (PIC <0.25 ). The g.414050897G > C locus of *GUCY1A1* showed moderate polymorphism in Small-tailed Han sheep ( 0.25< PIC <0.5 ) and low polymorphism in Hu sheep, Cele black sheep, Sunite sheep, and Bamei mutton sheep (PIC <0.25 ). The chi-squared test results showed that the g.405442728A > G locus of the *HRG* gene was in the Hardy–Weinberg equilibrium state in five sheep breeds ( P>0.05 ). The g.421655951C > T locus of the *FETUB* gene and the g.414050897G > C locus of the *GUCY1A1* gene were in the Hardy–Weinberg equilibrium state in Small-tailed Han sheep ( P>0.05 ) and in the Hardy–Weinberg disequilibrium state in other sheep breeds ( P<0.05 ).

**Table 3:** Population genetic analyses of the *HRG*, *FETUB*, and *GUCY1A1* polymorphisms in different sheep breeds.

Locus	Breed	Genotype frequency			Allele frequency		PIC	He	Ne	P
*HRG*		GG	GA	AA	G	A				
g.405442728A > G	Small-tailed Han sheep	0.02	0.16	0.82	0.43	0.57	0.37	0.49	1.96	0.51
	Hu sheep	0.01	0.22	0.77	0.40	0.60	0.36	0.48	1.92	0.51
	Cele black sheep	0.35	0.47	0.18	0.44	0.56	0.37	0.49	1.97	0.52
	Sunite sheep	0.02	0.23	0.75	0.40	0.60	0.36	0.48	1.92	0.44
	Bamei mutton sheep	0.10	0.38	0.52	0.36	0.64	0.36	0.46	1.86	0.55
		CC	CT	TT	C	T				
*FETUB*	Small-tailed Han sheep	0.86	0.13	0.01	0.93	0.07	0.12	0.13	1.15	0.30
g.421655951C > T	Hu sheep	0.98	0.02	0	0.99	0.01	0.02	0.02	1.02	0.00
	Cele black sheep	0.99	0.01	0	0.99	0.01	0.01	0.01	1.01	0.00
	Sunite sheep	0.89	0.11	0	0.95	0.05	0.09	0.10	1.11	0.00
	Bamei mutton sheep	0.91	0.09	0	0.95	0.05	0.09	0.09	1.10	0.00
		GG	GC	CC	G	C				
*GUCY1A1*	Small-tailed Han sheep	0.18	0.79	0.03	0.57	0.43	0.37	0.49	1.96	0.81
g.414050897G > C	Hu sheep	0.90	0.09	0.01	0.94	0.06	0.10	0.11	1.12	0.01
	Cele black sheep	0.90	0.10	0	0.95	0.05	0.09	0.10	1.11	0.00
	Sunite sheep	0.96	0.04	0	0.98	0.02	0.04	0.04	1.04	0.00
	Bamei mutton sheep	0.98	0.02	0	0.99	0.01	0.02	0.02	1.02	0.00

Note: monotocous: Sunite and Bamei mutton sheep; polytocous: Small-tailed Han sheep, Hu sheep, and Cele black sheep; PIC: polymorphic information content; He: heterozygosity; Ne: number of effective alleles.

### 3.2 Association analysis of *HRG*, *FETUB*, and *GUCY1A1* polymorphism and litter size of Small-tailed Han sheep

The association analysis between the genotypes of the three polymorphic loci of the *HRG*, *FETUB*, and *GUCY1A1* genes and the litter size of three parities of Small-tailed Han sheep was carried out. There was a significant correlation between the polymorphism of the g.405442728A > G locus of *HRG* and the litter size of the first and third parities of Small-tailed Han sheep ( P<0.05 ). The litter size of GG-type sheep in the first parity was significantly higher than that of the GA and AA types, and the litter size of GG-type sheep in the third parity was significantly lower than that of the AA type; there was a significant correlation between the polymorphism of the g.421655951C > T locus of *FETUB* and the litter size of the third parity of Small-tailed Han sheep ( P<0.01 ). In the third parity, the litter size of CT-type sheep was significantly lower than that of CC-type sheep. The mutation of the *GUCY1A1* gene had no significant influence on the litter size ( P>0.0 5) (Table 4).

**Table 4:** Association analysis of *HRG*, *FETUB*, and *GUCY1A1* polymorphism and litter size of Small-tailed Han sheep.

Locus	Genotype	Litter size of the first parity		Litter size of the second parity		Litter size of the third parity	
		Number	Litter size	Number	Litter size	Number	Litter size
*HRG*	GG	5	3.00 ± 0.32 ${}^{a}$	7	2.43 ± 0.20	2	1.50 ± 0.50 ${}^{a}$
g.405442728A > G	GA	60	2.12 ± 0.10 ${}^{b}$	43	2.65 ± 0.12	17	2.65 ± 0.23 ${}^{\mathrm{ab}}$
	AA	281	2.22 ± 0.05 ${}^{\mathrm{bc}}$	219	2.42 ± 0.06	79	3.01 ± 0.12 ${}^{b}$
*FETUB*	CC	288	2.18 ± 0.05	232	2.41 ± 0.06	76	2.68 ± 0.09 ${}^{A}$
g.421655951C > T	CT	49	2.33 ± 0.14	31	2.68 ± 0.18	11	2.45 ± 0.28 ${}^{B}$
	TT	2	2.50 ± 0.50	2	2.00 ± 0.00	2	2.50 ± 0.50 ${}^{\mathrm{AB}}$
*GUCY1A1*	GG	268	2.22 ± 0.05	214	2.46 ± 0.06	76	3.00 ± 0.12
g.414050897G > C	GC	66	2.21 ± 0.10	46	2.39 ± 0.16	19	2.73 ± 0.21
	CC	11	2.18 ± 0.23	9	2.67 ± 0.24	2	2.50 ± 0.50

Notes: the different uppercase letters indicate a highly significant degree ( P <0.01), and the different lowercase letters indicate a significant degree ( P <0.05).

### 3.3 Amino acid predictions of the *HRG*, *FETUB*, and *GUCY1A1* genes

To further study the effect of mutation on the structure of genes, the sequence of *HRG*, *FETUB*, and *GUCY1A1* before and after the mutation was used for amino acid prediction (Fig. 2). The minimum free energy of *HRG*, *FETUB* and *GUCY1A1* before and after mutation was -416.30 , -416.20 , -338.70 , -335.90 , -603.80 , and -602.90 kcal mol ${}^{-1}$ , respectively. The free energy of a spontaneous process will be reduced, so the transformation process of the above three genes can be carried out spontaneously.

## 4 Discussion

Any mutation of the gene locus is closely related to the role of the gene in the body. This article only involves one aspect for subsequent reference.

*HRG* seems to be an adaptive-molecule, histidine-rich glycoprotein (*HRG*) in plasma that can inhibit S100A8/A9-mediated melanoma cell organ metastasis, and plasma protein *HRG* plays a significant role in protecting the brain and lungs from melanoma metastasis (Tomonobu et al., 2022). *HRG* is also involved in tumor growth regulation (Karrlander et al., 2009; Tugues et al., 2012; Pan et al., 2022). *HRG* is a multi-domain molecule that interacts with a variety of gametes. The multi-domain structure of *HRG* indicates that the molecule can act as an adaptor protein to aggregate different ligands under certain conditions to play different roles (Jones et al., 2005). A preliminary study found that *HRG* is closely related to the maintenance and establishment of pregnancy (Nordqvist et al., 2011), and the gene exists in the structure of the female reproductive tract (Nordqvist et al., 2010). The existing results show that *HRG* is an important mammary gland mitogen for lactation (Li et al., 2002) and plays an important role in reproduction. Follicular fluid is rich in histidine glycoprotein. If its concentration in follicular fluid is lower than 35.80 mg dL ${}^{-1}$ , the possibility of obtaining live birth is greatly reduced (Zhang et al., 2021). Tsuchida Straten et al. (2005) reported that mice with *HRG* deletion can survive and reproduce without obvious abnormalities, but in some cases *HRG* deletion will have a greater impact (Wakabayashi, 2013). Our findings showed that the g.405442728A > G locus of the *HRG* gene had a significant impact on the litter size of Small-tailed Han sheep ( P<0.05 ). This indicates that the *HRG* gene can be used as a potential molecular marker for the litter size of Small-tailed Han sheep, which is consistent with the previous conclusion that the *HRG* gene is closely related to reproduction (Nordqvist et al., 2011; Lindgren et al., 2013).

Fetuin-B is a newly discovered member of the cystatin cysteine protease inhibitor superfamily (Jung et al., 2015; Meex et al., 2015). Studies have found that *FETUB* is related not only to coronary artery disease (Zhu et al., 2017) but also to reproduction, zona pellucida sclerosis, and sperm protein, thus affecting pregnancy and fertility. Reproduction is a complex process that is affected by many processes. Metabolic stability is a very important factor affecting sheep reproduction, and *FETUB* is an energy metabolism gene (Kralisch et al., 2017). Some studies have shown that the level of *FETUB* significantly increased during pregnancy (Simjak et al., 2018) (which may be related to the mechanism of pregnancy protection), endometrial translocation will lead to decreased fertility, and *FETUB* is related to this process (Cao et al., 2022). One study in mice found that *FETUB* is important for fertilization but is not so important for late pregnancy (Floehr et al., 2016). Stocker et al. (2014) found that *FETUB* maintains mammalian gamete fusion by inhibiting aspergillin, which is the switch triggering zona pellucida hardening. *FETUB* also plays a role in human reproduction, the *FETUB* content of women is higher than that of men, and estrogen or progesterone may be related to its regulation (Denecke et al., 2003). Studies have shown that the level of *FETUB* in plasma seriously affects the fertilization rate (Zhang et al., 2022). Our findings showed that the g.421655951C > T locus of the *FETUB* gene had a very significant impact on the litter size of Small-tailed Han sheep ( P<0.01 ). This result is consistent with the previous results, which proves that the *FETUB* gene plays an important role in animal reproduction.

Guanylate cyclase 1 soluble subunit α1 (*GUCY1A1*) can affect vascular reactivity and tubular function, thereby affecting thrombosis and leading to coronary artery disease (Malinowski et al., 2022). The SNP marker of *GUCY1A1* can also be used for the reproduction of Lufan black sheep (Liu et al., 2019). Studies have confirmed that the g.43266624C > T locus of the *GUCY1A1* gene is significantly related to the litter size in the field sheep (Ma et al., 2019). This study found that the g.414050897G > C locus of the *GUCY1A1* gene had no significant impact on the litter size of Small-tailed Han sheep ( P<0.05 ). The results of this experiment showed that there was no significant correlation between this locus and sheep reproduction, and the specific relationship needs to be further verified, which also provides a reference for further study of the role of the *GUCY1A1* gene.

## 5 Conclusions

In this study, the g.405442728A > G locus of the *HRG* gene exhibited moderate polymorphism ( 0.25< PIC <0.5 ) in both monotocous and polytocous sheep breeds, indicating that this locus has great selection potential in the five populations. Due to the existence of genetic variation, the above conclusion may not be desirable. However, among all the breeds tested, only Small-tailed Han sheep had a significant relationship between litter size and genotype. Therefore, it is accurate to say that the *HRG* gene can be used as a molecular marker to increase litter size in Small-tailed Han sheep. In Small-tailed Han sheep, the g.405442728A > G locus of the *HRG* gene and the g.421655951C > T locus of the *FETUB* gene had a significant effect on the litter size ( P<0.05 ), while the g.414050897G > C locus of the *GUCY1A1* gene had no significant effect ( P>0.05 ). Therefore, we can use the *HRG* and *FETUB* genes as candidate genes for the selection of litter size in sheep breeding.

## Data Availability

No data sets were used in this article.
